# Factors associated with modern contraceptive use among sexually active youths attending secondary schools in Mbale City, Uganda

**DOI:** 10.1371/journal.pgph.0005122

**Published:** 2025-10-03

**Authors:** Mary Abwola Olwedo, Nelson Bunani

**Affiliations:** 1 Soroti University, School of Health Sciences, Soroti, Uganda; 2 Makerere University School of Public Health, Kampala, Uganda; Babcock University, NIGERIA

## Abstract

Sexual and reproductive health of youths constitutes a significant public health challenge because of the high risk for morbidity and mortality. There is low uptake of modern contraceptives among the sexually active youths despite availability. We investigated the factors associated with the uptake of modern contraceptives among youths attending secondary schools in Mbale City. This was a cross-sectional study that enrolled 2,690 students from six purposively selected high-volume secondary schools in Mbale City. Data were collected using a validated semi-structured questionnaire and analyzed using descriptive statistics and multivariable logistic regression to identify factors associated with modern contraceptive use. The factors associated with modern contraceptive were stratified by sex. Statistical significance was set at 5% confidence level. Out of 2690 participants, 38.0% were sexually active. The proportion of sexually active participants who had ever used modern contraceptives was 60.9%. Factors associated with modern contraceptive use among male participants were not knowing the fertility days of the female (AOR = 0.49; 95% CI: 0.32-0.77; p = 0.002) and not receiving health education from a health worker (AOR = 0.44; 95% CI: 0.30-0.64; p < 0.001). Among females, factors associated with modern contraceptive use were knowledge of fertility days (AOR = 0.39; 95% CI: 0.18-0.85; p = 0.018), history of abortion (AOR = 0.10; 95% CI: 0.02-0.62; p = 0.014), and receiving health education from a health worker (AOR = 0.36; 95% CI: 0.22-0.59; p < 0.001). Modern contraceptive use was low compared to the national average, with knowledge of fertility days and health education significantly influencing uptake. Among males, lack of fertility knowledge and absence of health education were associated with low use of modern contraceptives. Among females, knowledge of fertility, history of abortion, and health education were key factors. Strengthening school-based sexuality education and increasing health worker-led reproductive health counselling can improve modern contraceptive knowledge and uptake, with gender-specific interventions needed to address barriers to access and use.

## Introduction

The global youth population, defined as individuals aged 15–24 years, currently stands at 1.3 billion and is projected to rise to 1.4 billion by 2030 [1]. Africa is home to an estimated 230 million youth in this age group, and this number is projected to increase by 42% in 2030 [2]. In Uganda, youth account for about 21.2% of the total population, making them a significant demographic group [3]. This group represent highly productive and energetic stages of life, often marked by risky behaviors such as exploratory sexual practices that expose them to unwanted pregnancies and sexually transmitted diseases [4,5]. Moreover, it has been estimated that 21 million young women became pregnant globally in 2020, the majority of whom were from low and middle-income countries (LMICs), mostly in sub-Saharan Africa (SSA), and alarmingly, nearly 50% of these pregnancies were unintended [6]. The high number of pregnancies is largely attributed to low contraceptive use [7].

The use of contraception among young women not only reduces pregnancy-related health risks [8] but also cuts the risk of other poor health outcomes for both the baby and the mother, including neonatal and maternal mortality [9]. However, SSA is affected by low contraceptive use [10,11]. Available data has revealed that only a third of the 11.3 million youth who need contraception have access [7,12].

Although data in East Africa has shown an increase in contraceptive use among women of reproductive ages [11], unmet needs remain disproportionately high in Uganda, particularly among sexually active youth [13]. In Uganda, 42% of pregnancies among youth are unintended, and 24% of women aged 15–19 have begun childbearing, with the eastern region, including Mbale, bearing a significant burden [13]. Early pregnancies have far-reaching social and educational consequences, with 34% of pregnant schoolgirls dropping out of school and many facing stigma that prevents them from returning [14].

The onset of sexual activity in Uganda and other parts of SSA occurs between the ages of 15.8 and 25.37 years, often overlapping with school attendance [15,16]. However, there is limited data on modern contraceptive use among school-attending youth despite their increased vulnerability to unintended pregnancies and associated consequences [17–19]. Fertility awareness, specifically, knowledge of when a girl is most likely to conceive is a critical component of reproductive health knowledge. Adolescents who understand the concept of unsafe days during the menstrual cycle are better positioned to make informed choices about contraceptive use.

In selecting explanatory variables, we drew on empirical literature and reproductive health frameworks to ensure relevance to modern contraceptive use. One such variable is knowledge of fertility or unsafe days, that is, awareness of when a girl is most likely to become pregnant during her menstrual cycle. This variable reflects a youth’s level of fertility awareness, which has been shown to influence reproductive decision-making and contraceptive behavior [20,21]. Adolescents who lack accurate knowledge about fertility often underestimate their pregnancy risk, leading to low or inconsistent contraceptive use [7,20]. In contrast, those who are aware of the fertile window are more likely to recognize the consequences of unprotected sex and to adopt preventive measures, including the use of modern contraceptives [21].

Previous studies in sub-Saharan Africa and globally have confirmed the positive association between fertility knowledge and contraceptive uptake among adolescents and young women [21–23]. Including this variable in our instrument allowed us to assess whether such awareness is a significant factor influencing contraceptive behavior among sexually active school-going youths in Uganda, particularly in a context where comprehensive sexuality education may be limited or inconsistently delivered [24,25]. This study sought to determine the prevalence of modern contraceptive use and associated factors among sexually active youths aged 15–24 years attending secondary schools in Mbale City, Uganda.

## Materials and methods

### Study design, setting, and population

This cross-sectional study was conducted among youths aged 15–24 years, purposively selected from high-volume secondary schools in Mbale City to ensure a representative sample. The study was conducted from 15^th^ June to 17^th^ July 2017. According to data from the Mbale City Education Office, the city has 31 secondary schools, both government and privately owned. The study included Nkoma Senior Secondary School, Mbale Senior Secondary School, Bugisu High School, Oxford, Wanale View, and Mbale High School. Mbale City was purposively selected as it is part of Mbale District in the Elgon region, where sexual debut occurs as early as 13 years among girls and 12 years among boys, and the fertility rate is high [16,26]. Additionally, the district practices traditional circumcision, an activity associated with illicit sex and has highest teenage pregnancy rates, a major contributor to school dropouts in Uganda [26]. Only students from Senior Two to Senior Six who had attended their respective schools for at least one term were included. The youths aged 15–24 years were selected because this age group is reported to be vulnerable to early sexual debut and reproductive health risks in Uganda, with the age of first sexual activity commonly ranging between 15 and 17 years [16,27]. The UDHS 2022 further revealed that 23.5% of women aged 15–19 had initiated childbearing.

### Sample size and sampling procedure

Using the Kish Leslie formula [28], a prevalence of 62% [29], a design effect of 2, and a response rate of 90%, with an estimated 30% of secondary school students being sexually active, a total sample size of 2,690 students was determined.

A multistage sampling approach was used. Six high-volume secondary schools were purposively selected based on student population size per school. Using probability proportionate to size, the total sample of 2,690 students was distributed as follows: Nkoma Senior Secondary School (938 students), Mbale Secondary School (858), Oxford Secondary School (402), Mbale High School (268), Bugisu High School (134), and Wanale View Secondary School (80). Within each school, the allocated sample was proportionately divided across five class levels (Senior Two to Senior Six) and further stratified by the number of available streams per class.

Systematic random sampling was then used to select students within each stream. The sampling interval (k) was determined by dividing the total number of eligible students in a stream by the number of participants required. A random starting point was identified by selecting one of the students seated near the classroom door, and every kth student was selected until the desired sample was obtained. This process was repeated across all streams and class levels until the computed sample size was attained.

### Study variables

The dependent variable was modern contraceptive use which was measured as a binary outcome. This was assessed using the question: “Have you ever had sexual intercourse?” A “Yes” response indicated sexual activity. Those who were sexually active were further asked, “Have you ever used modern contraceptives?” Respondents who answered “Yes” were coded as 1, while those who answered “No” were coded as 0. Independent variables included religion, categorized as 1 = Catholic, 2 = Anglican, 3 = Born Again, and 4 = Others (including Seventh-Day Adventists, Orthodox, and Traditionalists). Place of residence was categorized as 1 = Residing in a hostel, 2 = Residing in a rented room, and 3 = Commuting from home. Age was collected as a continuous variable and categorized during analysis. Sex was categorized as 1 = Male and 2 = Female. The number of sexual partners was collected as a continuous variable and categorized during analysis. Ever impregnated a girl was assessed among male respondents and categorized as 1 = Yes and 2 = No. Received health education was categorized as 1 = Yes and 2 = No. Knowledge of a girl’s fertility days was categorized as 1 = During menstruation, 2 = One week after menstruation, 3 = Two weeks after menstruation, and 4 = Don’t know.

### Data collection

The identified study participants were assessed whether they resided in the school hostel or at home with their guardians; those in the school hostel, the head teacher consented for them to participate in the study before they assented to participate. Those aged 15–17 years residing at home with parents/guardians were given parent/guardian consent forms, and they participated in the study the next day when they presented signed consent from their parents/guardians. A semi-structured questionnaire was used to collect the data. The tool was administered to the study participants through face-to-face interviews. Before data collection, the tool was pretested on the students in one of the schools, which was not part of the study sites, to assess whether the questions were valid to measure the intended purpose. Based on data from the pretest, some questions were rephrased to ensure that they collect the intended data.

### Data management and analysis

Data were entered into excel, checked for completeness, cleaned, and exported into SPSS version 16 and STATA version 12 for analysis. Descriptive statistics were used to present the participants’ background characteristics, while logistic regression was used in bivariate and multivariable analysis to identify factors associated with modern contraceptive use among youths attending secondary schools in Mbale city. Associations were tested at a 95% confidence interval (CI). The factors which had a p-value <0.2 at bivariable analysis and those that were biologically plausible were included in the multivariable analysis model to obtain adjusted odds ratios. Model building was done by stepwise elimination, which involved adding the variables that qualified for multivariable level one at a time while dropping those with no significance. Unadjusted and adjusted odds ratios and their 95% confidence intervals (CIs) and p-values are reported.

## Results

### Sociodemographic characteristics of study participants

As shown in Table 1, 38.0% (1023/2690) of the participants reported ever having had sex, while 15.9% (163/1023) had ever been pregnant or impregnated. More than half, 60.9% (623/1023), had ever used contraceptives, and 42.5% (435/1023) had engaged in sexual activity in the last three months. Among schools, Nkoma senior secondary school had the highest proportion of students who had ever had sex, 45.0% (395/878), and had ever been pregnant or impregnated, 17.0% (67/395). Bugisu High had the highest proportion of students who had ever used contraceptives, 73.6% (53/72). Nkoma senior secondary school also had the highest proportion of students who had sex in the last three months, 46.3% (183/395). The same category had the highest proportion of those who had ever been pregnant or impregnated, 25.8% (23/89), and those who had ever used contraceptives, 65.2% (58/89). More than half, 53.9% (48/89), had had sex in the last three months.

**Table 1:** Sociodemographic characteristics of study participants.

	Total, n = 2690 (%)	Ever had sex, n = 1023 (%)	Ever been pregnant/impregnated a girl, n = 163 (%)	Ever used contraceptive, n = 623 (%)	Had sex in last 3 months, n = 435 (%)
**School**	**2690(100.0)**	**1023(38.0)**	**163(15.9)**	**623(60.9)**	**435(42.5)**
Bugisu High	149(5.5)	72(48.3)	11(15.3)	53(73.6)	31(43.1)
Mbale Senior Secondary School	872(32.4)	247(28.3)	40(16.2)	135(54.7)	106(42.9)
Mbale High	317(11.8)	129(40.7)	18(14)	85(65.9)	44(34.1)
Nkoma Senior Secondary School	878(32.6)	395(45.0)	67(17.0)	245(62.0)	183(46.3)
Oxford Senior Secondary School	390(14.5)	144(36.9)	24(16.7)	79(54.9)	61(42.4)
Wanale View Senior Secondary School	84(3.1)	36(42.9)	3(8.3)	26(72.2)	10(27.8)
**Religion**					
Catholic	657(24.4)	240(36.5)	35(14.6)	147(61.3)	99(41.3)
Anglican	539(20.0)	196(36.4)	29(14.8)	109(55.6)	89(45.4)
Moslem	626(23.3)	276(44.1)	49(17.8)	176(63.8)	116(42)
Born Again	672(25.0)	222(33.0)	27(12.2)	133(59.9)	83(37.4)
Others	196(7.3)	89(45.4)	23(25.8)	58(65.2)	48(53.9)
**Education Level**					
Senior two	433(16.1)	123(28.4)	27(22.0)	65(52.8)	55(44.7)
Senior three	785(29.2)	276(35.2)	39(14.1)	156(56.5)	112(40.6)
Senior four	646(24.0)	211(32.7)	28(13.3)	134(63.5)	82(38.9)
Senior five	339(12.6)	139(41.0)	20(14.4)	89(64.0)	62(44.6)
Senior six	487(18.1)	274(56.3)	49(17.9)	179(65.3)	124(45.3)
**Residence**					
Hostel	584(21.7)	215(36.8)	36(16.7)	136(63.3)	95(44.2)
Rented a room in the municipality	546(20.3)	274(50.2)	51(18.6)	171(62.4)	125(45.6)
Commuted from home	1560(58.0)	534(34.2)	76(14.2)	316(59.2)	215(40.3)
**Age**					
15-17	1345(50.0)	348(25.9)	59(17.0)	195(56.0)	141(40.5)
18-19	1000(37.2)	452(45.2)	62(13.7)	272(60.2)	195(43.1)
20-24	345(22.8)	223(64.6)	42(18.8)	156(70.0)	99(44.4)
**Sex**	2690(100.0)	1023(38.0)	163(15.9)	623(60.9)	435(42.5)
Male	1495(55.6)	702(47)	130(18.5)	444(63.2)	322(45.9)
Female	1195(44.4)	321(26.9)	33(10.3)	179(55.8)	113(35.2)

More than half of the Senior Six students, 56.3% (274/487), reported ever having had sex, and 17.9% (49/274) had ever been pregnant or impregnated. The highest proportion of contraceptive use, 65.3% (179/274), was also among Senior Six students, while 45.3% (124/274) had engaged in sexual activity in the last three months. The students who rented a room in the municipality had the highest proportion of those who had ever had sex, 50.2% (274/546), and ever been pregnant or impregnated, 18.6% (51/274). More than three out of five, 63.3% (136/215), of those in hostels had ever used contraceptives, while 45.6% (125/274) of those who rented a room in the municipality had sex in the last three months.

A higher proportion of students who had ever had sex was in the 20–24 age group, 64.6% (223/345), and 18.8% (42/223) had ever been pregnant or impregnated. Seven in ten, 70.0% (156/223), had ever used contraceptives, while 44.4% (99/223) had engaged in sexual activity in the last three months. Males had a higher proportion of those who had ever had sex, 47.0% (702/1495), compared to females at 26.9% (321/1195). More males, 18.5% (130/702), had ever been involved in a pregnancy, compared to 10.3% (33/321) of females. More than three in five males, 63.2% (444/702) had ever used contraceptives, compared to 55.8% (179/321) of females. Additionally, 45.9% (322/702) of males had engaged in sexual activity in the last three months, compared to 35.2% (113/321) of females.

### Prevalence of modern contraceptive use among sexually active students

As shown in Table 2, more than half of the sexually active students, 60.9% (623/1023) had used modern contraception methods, with 63.2% (444/702), of whom were male students. Nearly a third, 26.5% (271/1023) of the respondents were currently on contraceptives at the time of the study.

**Table 2:** Prevalence of modern contraceptive use among sexually active students.

	Ever used modern contraceptives	
**Sex**	**No (%)**	**Yes (%)**
Male	258 (36.8)	444 (63.2)
Female	142 (44.2)	179 (55.8)
**Total**	**400 (39.1%)**	**623 (60.9)**
	**Currently on modern contraceptives or girlfriends are** **No (%) Yes (%)**	
Male	505 (71.9)	197 (28.1)
Female	247 (76.9)	74 (23.1)
**Total**	**752(73.5)**	**271(26.5)**

### Factors associated with modern contraceptive use among sexually active males

As shown in Table 3, at bivariate analysis, being 20–24 years, attaining Senior Six education, having multiple sexual partners, not knowing the girl’s fertility days, never impregnating a girlfriend before, never having an abortion, and not receiving health education from a health worker were significantly associated with modern contraceptive use. The odds of modern contraceptive use among males aged 20–24 years were 1.99 times the odds among those aged 15–17 years (OR=1.99; 95% CI: 1.29-3.07; *p* = 0.002). Males with Senior Six education had 2.12 times the odds of modern contraceptive use compared to those with Senior Two education (OR=2.12; 95% CI: 1.26-3.54; *p* = 0.004). Those with two or more sexual partners had 1.49 times the odds of modern contraceptive use compared to those with only one partner (OR=1.49; 95% CI: 1.06-2.01; *p* = 0.020). Furthermore, males who did not know the girl’s fertility days had 0.50 times the odds of modern contraceptive use compared to those who thought fertility occurred during menstruation (OR=0.50; 95% CI: 0.33-0.76; *p* = 0.001). The odds of modern contraceptive use were 0.52 times among those who had never impregnated a girlfriend compared to those who had (OR=0.52; 95% CI: 0.34-0.81; *p* = 0.003). The odds were 0.49 times among those who had never had an abortion compared to those who had (OR=0.49; 95% CI: 0.27-0.87; *p* = 0.016). Those who never received health education from a health worker had 0.39 times the odds of modern contraceptive use compared to those who had received it (OR=0.39; 95% CI: 0.27-0.56; *p* < 0.001).

**Table 3:** Factors associated with modern contraceptive use among sexually active male students in schools in Mbale city.

Variable	Number of Observation	Bivariate			Multivariable		
		UOR	95% CI	P-Value	AOR	95% CI	P-Value
**Age (Years)**	**702**						
15-17	224	1			1		
18-19	309	1.15	[0.81-1.64]	0.422	0.90	[0.57-1.40]	0.633
20- 24	169	1.99	[1.29-3.07]	**0.002** ^*^	1.43	[0.79-2.57]	0.234
**Education**	**702**						
Senior two	95	1			1		
Senior three	177	1.03	[0.62-1.70]	0.908	1.20	[070-2.05]	0.516
Senior four	131	1.55	[0.90-2.66]	0.112	1.74	[0.93- 3.27]	0.083
Senior five	114	1.44	[0.83-2.52]	0.194	1.42	[0.72-2.82]	0.314
Senior six	185	2.12	[1.26-3.54]	**0.004** ^*^	1.69	[0.85-3.33]	0.132
**Number of sexual partners**	**702**						
1	420	1					
>=2	282	1.49	[1.06-2.01]	**0.020** ^*^	1.40	[0.99-1.96]	0.054
**Time at which the girls get to know about unsafe days**	**702**						
During menstruation	146	1			1		
1 week after menstruation	184	1.23	[0.77-1.99]	0.390	1.06	[0.64 -1.75]	0.817
2 weeks after menstruation	103	0.98	[0.57-1.68]	0.929	0.77	[0.43-1.37]	0.376
I don’t know	269	0.50	[0.33-0.76]	**0.001** ^*^	0.49	[0.32-0.77]	**0.002** ^*^
**Ever Impregnated girlfriend**	**702**						
Yes	130	1			1		
No	572	0.52	[0.34 -0.81]	**0.003** ^*^	0.66	[0.35-1.23]	0.189
**Ever aborted**	**702**						
Yes	69	1			1		
No	633	0.49	[0.27 -0.87]	**0.016** ^*^	0.85	[0.37-1.94]	0.696
**Received health education from Health workers**	**702**						
Yes	207	1			1		
No	495	0.39	[0.27 -0.56]	**<0.001** ^*^	0.44	[0.30-0.64]	**<0.001** ^*^

*P-value is significant at ≤ 0.05, UOR- Unadjusted odds ratio, AOR-Adjusted odds ratio, CI-Confidence interval.

In multivariable analysis, not knowing the girl’s fertility days and not receiving health education from a health worker remained significantly associated with contraceptive use. The odds of modern contraceptive use among males who did not know the girl’s fertility days were 0.49 times the odds among those who thought fertility occurred during menstruation (AOR = 0.49; 95% CI: 0.32-0.77; *p* = 0.002). Similarly, the odds of modern contraceptive use among those who never received health education from a health worker were 0.44 times the odds among those who had received it (AOR = 0.44; 95% CI: 0.30-0.64; *p* < 0.001).

### Factors associated with modern contraceptive use among sexually active females

Table 4 shows that in bivariate analysis, never been pregnant, not knowing the girl’s fertility days, never having an abortion and not receiving health education on family planning from a health worker, were negatively associated with modern contraceptive use. The odds of contraceptive use among those who had never been pregnant were 0.44 times the odds among those who had been pregnant (OR=0.44; 95% CI: 0.20-0.97; *p* = 0.043). The odds of modern contraceptive use among those who did not know the girls’ fertility days were 0.47 times the odds among those who believed fertility occurred during menstruation (OR=0.47; 95% CI: 0.22-0.98; *p* = 0.043). The odds of modern contraceptive use among those who had never had an abortion were 0.22 times the odds among those who had an abortion (OR=0.22; 95% CI: 0.07-0.65; *p* = 0.006). The odds of modern contraceptive use among those who never received health education on family planning were 0.33 times the odds among those who had received health education (OR=0.33; 95% CI: 0.21–0.54; *p* < 0.001).

**Table 4:** Factors associated with modern contraceptive use among sexually active female students in schools in Mbale city.

Variable	Number of Observations	Bivariate			Multivariate		
		UOR	95% CI	P-Value	AOR	95% CI	P-Value
**Ever been pregnant**	**321**						
Yes	33	1			1		
No	288	0.44	[0.20-0.97]	0.043*	2.60	[0.57- 11.85]	0.216
**Time at which the girls got to know about unsafe days**	**321**						
During menstruation	46	1			1		
1 week after menstruation	115	1.00	[0.50-2.01]	1.000	0.84	[0.40-1.75]	0.638
2 weeks after menstruation	77	0.95	[0.45-2.01]	0.901	0.81	[0.37-1.78]	0.605
I don’t know	83	0.47	[0.22-0.98]	0.043*	0.39	[0.18-0.85]	0.018*
**Ever Aborted**	**321**						
Yes	25	1			1		
No	296	0.22	[0.07-0.65]	0.006*	0.10	[0.02-0.62]	0.014*
**Health education on FP by health worker**	**321**						
Yes	112	1			1		
No	209	0.33	[0.21 – 0.54]	<0.001^*^	0.36	[0.22-0.59]	**<0.001** ^*^

*P-value is significant at ≤ 0.05, UOR- Unadjusted odds ratio, AOR-Adjusted odds ratio, CI-Confidence interval.

In the multivariable model, knowledge of fertility days, abortion history, and health education were significantly associated with modern contraceptive use. The odds of modern contraceptive use among those who did not know the girl’s fertility days were 0.39 times the odds among those who thought fertility occurred during menstruation (AOR = 0.39; 95% CI: 0.18-0.85; *p* = 0.018). The odds of modern contraceptive use among those who had never had an abortion were 0.10 times the odds among those who had an abortion (AOR = 0.10; 95% CI: 0.02-0.62; *p* = 0.014). The odds of modern contraceptive use among those who never received health education on family planning were 0.36 times the odds among those who had received health education (AOR = 0.36; 95% CI: 0.22-0.59; *p* < 0.001).

## Discussion

This study aimed to determine the prevalence of modern contraceptive use and associated factors among sexually active youths aged 15–24 years attending secondary schools in Mbale municipality. The prevalence of modern contraceptive use was 60.7% (71.3% among males, 28.7% among females). This prevalence was far higher than that reported by previous studies [30–32]. The higher prevalence is attributed to the free sexual and reproductive health services provided by the youth/adolescent-friendly services at Mbale Regional Referral Hospital, which is the main health facility in the area. The schools are also located in Mbale, which allows the youths to access information on media easily. The Uganda national family planning policy also allows youths to access contraceptive methods without parental consent; this, therefore, encourages the youths to access contraceptive methods [33]. Furthermore, Mbale district, a traditionally circumcising community, experiences a surge in illicit sexual activity during circumcision seasons, held every other year. To avoid unintended pregnancies while continuing their education, many youths engaged in such activities are likely motivated to use contraceptives.

Despite the relatively high prevalence, gender disparities in contraceptive use were observed, with males reporting higher use than females. Among male participants, lack of knowledge about the girl’s fertility days was associated with significantly lower odds of contraceptive use. This finding aligns with previous studies from different parts of Sub-Saharan African settings, which have shown that a limited understanding of fertility cycles contributes to reduced contraceptive use [20,21]. Poor awareness of fertility periods can lead to misconceptions about pregnancy risks, thereby reducing motivation to use contraceptives [21]. These findings highlight the need for comprehensive sexuality education to improve knowledge and foster male engagement in reproductive health decision-making.

Furthermore, not receiving family planning education from a health worker was significantly associated with lower contraceptive use among males. This underscores the crucial role of healthcare providers in delivering accurate reproductive health information [34]. Related findings have been reported in Uganda and other sub-Saharan Africa [35,36]. These findings reinforce the importance of integrating health worker-led reproductive health education into school curricula to bridge knowledge gaps and improve informed contraceptive choices among young men.

Among female participants, a history of abortion was strongly associated with contraceptive use, suggesting that previous pregnancy experiences increased motivation to use contraception to avoid unintended pregnancies. Comparably, a study in Angola found that a history of abortion had a higher probability of using a modern method [37]. This finding emphasizes the importance of early contraceptive education, ensuring that young women have access to information before experiencing unintended pregnancies.

Additionally, not receiving health education on family planning from a health worker was linked to lower contraceptive use among females, a finding that mirrors the results among males. This reinforces the significance of health worker-led education in promoting contraceptive uptake [34]. Previous studies in Uganda and elsewhere have also found that limited health education, which limits information on family planning, has been associated with low contraceptive use [38,39]. Expanding school-based reproductive health counselling programs and increasing access to youth-friendly services near schools could improve uptake, particularly among female students.

The study also found that lack of knowledge about fertility periods significantly reduced contraceptive use among females, similar to its impact on males. A possible explanation is irregular school attendance, which prevents students from receiving reproductive health lessons during classes, counselling sessions, and career guidance programs [22,40–42]. Our findings suggest a need to strengthen school-based sexuality education, and ensuring consistent participation in reproductive health sessions may improve contraceptive knowledge and utilization.

## Strengths and limitations

### Study limitations

The study employed a large sample size using multi-stage sampling, which ensured representativeness across different schools and socio-demographic groups, enhancing the generalizability of the findings. However, self-reported data using a questionnaire may have led to social desirability and recall bias, potentially affecting the accuracy of responses on contraceptive use and sexual activity. This was addressed by training the research assistants to ask the questions appropriately and get correct responses.

The study did not incorporate a theoretical framework to guide the selection of predictor variables, which may limit the conceptual rigour of the analysis. However, this was addressed through ensuring that variable selection was grounded in empirical evidence, previous studies, and contextual relevance to the target population.

## Conclusion and implications

Among the 15–24-year-old youths attending six secondary schools in Mbale City, 38.1% were sexually active, 60.7% had ever used modern contraceptive methods, and 26.5% were currently using modern contraceptives. Male students were more likely to report modern contraceptive use than females. Whereas never aborted was associated with low modern contraceptive use among the girls only. Lack of knowledge of girl’s fertility days in the menstrual cycle and lack of health education on family planning by health workers were associated with low odds of modern contraceptive use among both male and female respondents respectively. Health facilities were the main source of modern contraceptive methods, and condoms were the most common method used. The findings underscore the need for targeted interventions that improve knowledge of fertility, provide health worker led reproductive education and address gender specific barriers to modern contraceptive and among sexually active youths.

## Supporting information

S1 Data S1_Data (XLS)
